# Is it necessary to use a drain after urethroplastyby perineal approach if we can avoid complications by pressure dressing?

**DOI:** 10.1186/s12894-025-01768-w

**Published:** 2025-05-19

**Authors:** Ege A. Sarıkaya, Sümeyye Terzi, Atahan Çakmak, M. Selçuk Özer, Volkan Şen, Ozan Bozkurt

**Affiliations:** DokuzEylul University Hospital Urology Department, Izmir, Turkey

**Keywords:** Urethroplasty, Perineal drainage, Urethral stricture

## Abstract

**Purpose:**

Peri-operative management of urethroplasty is yet to be standardized. One of the major obscure issues in perioperative management is the use of perineal drainage. Some reconstructive urologists prefer to use a drain as a routine while others never use one. Although the main purpose of drain placement is to prevent the collection and related wound complications, no study up-to-date refers to these complications neither with nor without drain usage.

**Methods:**

152 consecutive patients who underwent urethroplastyvia perineal approach without perineal drain were included. Strict pressure dressing was applied to all patients. The presence of any perineal fluid collection, wound related complications and the need for re-operation for these complications were recorded.

**Results:**

Despite 42% of patients having a smoking history and other potential risk factors for wound related complications, there were no instances of fluid collection or wound dehiscence post-operatively.

**Conclusion:**

Given the absolute absence of drainage related complications by applying only pressure dressing, we suggest that a perineal drain is not necessary after urethroplasty via perineal approach. Applying pressure dressing is an effective method to prevent fluid collection and related morbidities.

## Introduction

Urethral stricture is one of the most common reasons of infravesical obstruction. Surgical intervention is mandatory and endourological approaches usually require repeated surgeries and eventually end up in failure. Urethroplasty on the other hand, has success rates as high as 80–90% and is considered to be the gold standard surgical approach for recurrent strictures [1, 2].

Depending on the stricture site, urethroplasty can be performed with a penile, perineal or abdominal incision. Penile incision is usually preferred for distal anterior strictures, while perineal incision is usually necessary for long segment strictures, posterior strictures, and proximal anterior strictures. Abdominal approach is usually reserved for strictures regarding bladder neck [3].

Multiple surgical urethroplasty methods are defined that can be performed with a perineal incision. Excision and anastomotic urethroplasty (EPA), dorsal inlay free-graft urethroplasty (FGU), dorsal onlay FGU, non-transecting EPA, ventral onlay FGU are few of the most well-defined and well-established surgical techniques. Although the steps of these surgical methods are well-established and almost identical in many clinics, there is no consensus regarding the placement of a drain after perineal incision. In penile incision, drain placement is usually not an option while a drain is almost always necessary in abdominal incision to prevent collections such as hematoma, urinoma, lymphocele and seroma that can eventually turn into pelvic abscess and impair wound healing. In perineal incision however, some urologists routinely insert a drainage catheter while others never use one [4].

Applying strict pressure dressing is an alternative method to prevent fluid collection and can replace drain placement after perineal incision. In our clinic, we routinely apply pressure dressing post-operatively and we never use a perineal drain. We aimed to present our method of dressing and post-operative results regarding wound healing and complications due to fluid collection in consecutive urethroplasty cases to prove the efficiency and safety of pressure dressing.

## Materials and methods

152consecutive patients who underwent urethroplasty by perineal approachbetween January 2018 and May 2024 were retrospectively examined. Characteristics of strictures and patients and surgical methods were examined. Penile and abdominal approaches were not included.

**Fig. 1:**
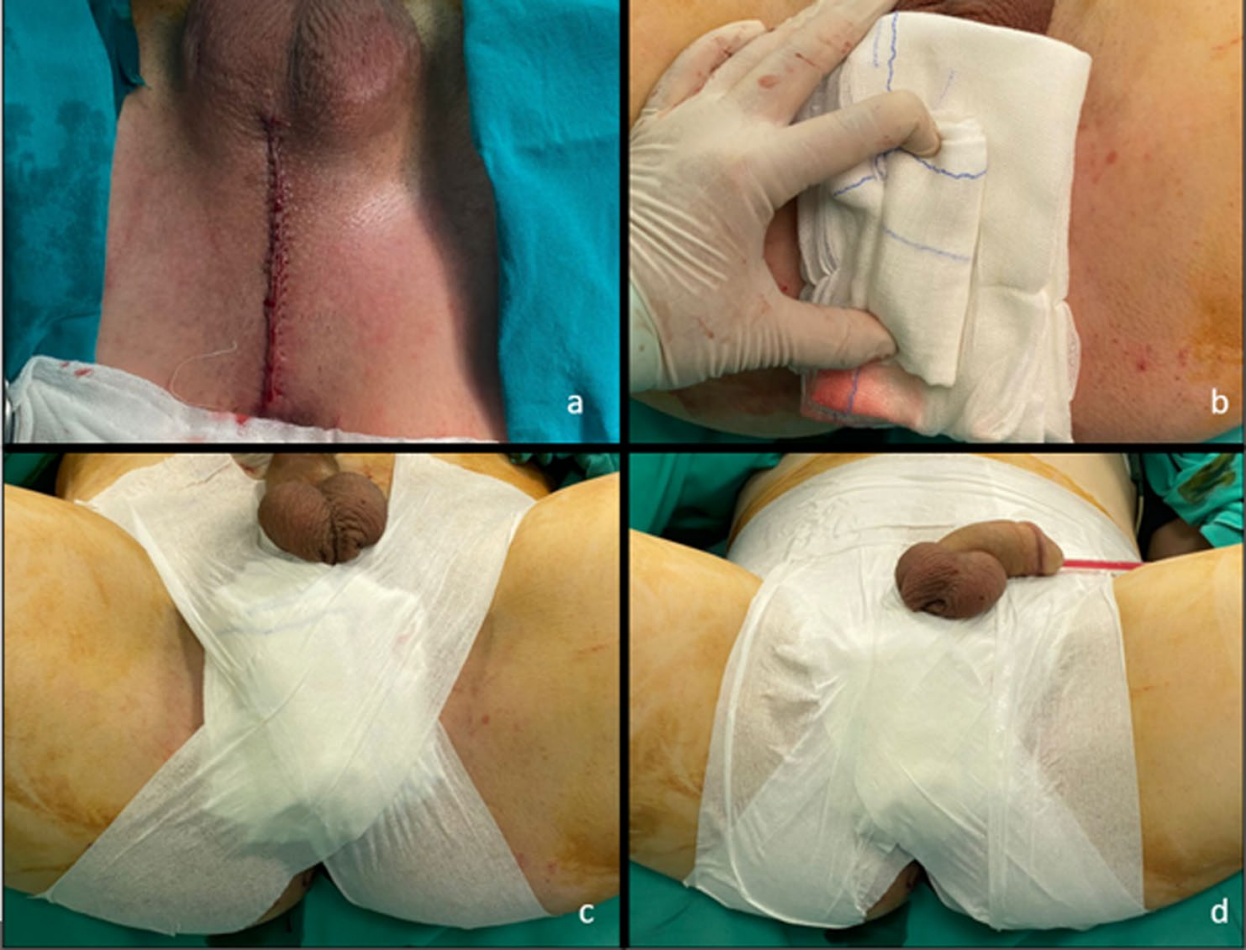
**(a)** Closure of perineal skin before applying strict pressure dressing, **(b)** Placement of three pads a tightly rolled pad over them to increase the pressure, (**c)** X-shaped bandage between the back of the thighs and the contralateral lower abdomen, (**d)** Horizontal and vertical bandages to secure strictness and prevent the loss of pressure

Negative urine cultures were obtained from all patients within a week of the operation. A perineal vertical incision was performed in all patients. Vertical incision of bulbospongiosus muscle at midline was performed ifnecessary according to the location of stricture. In all anastomotic urethroplasties for membranous and proximal bulbar strictures, complete mobilisation of corpus spongiosum and corporeal dissection of cavernosums were performed. Inferior pubectomy was also performed when necessary. After urethral mucosal repair over a 16 or 18 f urethral catheter (FGU or anastomotic), if a vertical urethrotomy had been performed, a 4/0 continuous monofilament suture was used for closure of corpus spongiosum. Bulbospongiosis muscle was repaired with a continuous 2/0 vicryl suture. Subcutaneous adipose tissue and buck’s fascia was closed with interrupted 3/0 sutures. The skin was closed with 4/0 continuous rapid vicryl and a drainage catheter was not inserted in any cases (Fig. 1a). Only bipolar electro-cauterization was used for bleeding control during all steps of operations. After wound closure a strict pressure dressing was applied to the perineum. As its first step; three pads were placed over the incision and a tightly rolled pad over them to increase the pressure (Fig. 1b). Then, an X-shaped bandage was applied between the back of the thighs and the contralateral lower abdomen with nearly 75 cm bandages in lithotomy position (Fig. 1c). Lastly, horizontal and vertical bandages were applied over to secure strictness and prevent the loss of pressure (Fig. 1d). Pressure dressing was controlled every six hours for two days post-operatively and removed after two days. Patients were only mobilized in bed on post-operative day 1 and fully mobilized on day 2. The presence of any perineal fluid, hematoma, urinoma, abscess, wound dehiscence and need for re-operation or antibiotic revision for these complications were recorded.

## Results

Patients’ and strictures characteristics and surgical methods are given in Table 1. Strict pressure dressing was applied to all patients and perineal drainage catheter was not used in any cases. 64 (42%) patients had a smoking history, 25 (16%) had obesity and seven (4%) had a history of pelvic radiation as a complicating factor that might impair wound healing. No patients were on antiaggregant or anti-coagulant treatment at the time of the operation.As a surgical manoeuvre that may aggravate post-operative bleeding and fluid collection; complete mobilisation of corpus spongiosum and corporal dissection of corpus cavernosums were performed in 30 cases and inferior pubectomy was performed in four cases. No patient had any kind of fluid collection or wound dehiscence post-operatively.10 patients had mild and transient ecchymosis around incision site which were all spontaneously healed and no adjacent intervention were needed.No fluid collection related complications such as hematoma, swelling, urinoma or seroma requiring surgical intervention were observed and no need for antibiotic revision for post-operative wound infection waspresent.

**Table 1:** Characteristics of patients and surgical methods

Age, Mean (Min-Max)	55,88 (18–87)
**Length of Hospital Stay**,** Mean** (Min-Max)	3–7 (3,3)
**Catheter Removal Time**,** Mean** (Min-Max / Days)	26 (14–42)
**Follow-up Duration**,** Mean** (Min-Max / Months)	32 (9–85)
**History of Smoking**,** n** (%)	64 (42,1)
**Obesity**,** BMI > 30**,n (%)	25 (16,4)
**History of Pelvic Radiation**,** n** (%)	7 (4,6)
**Etiology**,** n** (%)	
TUR-P	48 (31,6)
Urethral Catheterisation	32 (21)
Trauma	25 (16,4)
Other Transurethral Operations	17 (11,2)
Radiotherapy	6 (3,9)
Radical Prostatectomy	3 (2)
Urethritis	2 (1,3)
Idiopathic	19 (12,5)
**Localisation of Stricture**, n (%)	
Bulbomembranous	54 (35,5)
Membranous	34 (22,3)
Bulber	24 (15,8)
Penobulber	17 (11,2)
Panurethral	9 (5,9)
Proximal Penile	7 (4,6)
Multiple Segment	7 (4,6)
**Surgical Method**, n (%)	
Ventral Onlay FGU	89 (58,6)
Anastomotic Urethroplasty (EPA)	30 (19,7)
Dorsal Inlay + Ventral OnlayFGU	9 (5,9)
EPA + FGU	9 (5,9)
Dorsal Inlay FGU	8 (5,3)
DorsolateralOnlay FGU	4 (2,6)
Dorsal Onlay FGU	2 (1,3)
Non-transecting EPA	1 (0,7)

## Discussion

Urethroplasty is the only option to cure recurrent urethral strictures. With many surgical methods described depending on mainly stricture site and length, overall success rates are approximately 80–90%. Surgical steps of several different techniques such as dorsal inlay FGU, anastomotic FGU, dorsolateral onlay FGU, ventral onlay FGU, dorsal inlay FGU non-transecting anastomotic FGU are well defined and usually do not differ between surgeons. However, perioperative management of urethroplasty is not as homogenous. One of the issues in perioperative management that needs to be decided is the placement of a perineal drain. Some clinicians prefer to use a perineal drainage catheter as a routine for a day or two post-operatively while others only use pressure dressing to prevent any fluid collection [4].

A drain is mainly required for drainage of a fluid collection or to prevent one from happening. These fluid collections may be seroma, lymphocele, hematoma or urinoma which all can be a reason of discomfort or eventually lead to abscess and impair wound healing. In the site of the perineal midline incision, lymphatic circulation is extremely poor and the formation of a lymphocele is not a concern. Also, there is no physiological fluid circulation to cause seroma in the perineum like peritoneum or tunica vaginalis. After almost all urethroplasty surgeries a urethral catheter is inserted to prevent urine leakage, thus preventing urinoma.

The only likely possible fluid collection to cause complications after urethroplasty seems to be hematoma. To prevent adjacent injury, monopolar cauterization is not preferred in the perineal and penile area. Bipolar cauterization should be used and careful bleeding control should be done to minimize the risk for hematoma. However, the effectiveness of bipolar cauterization may not be as high as monopolar cauterization, and many urologists prefer to apply strict pressure dressing to enhance bleeding control and prevent hematoma.

We did not use perineal catheter in any of the 152 cases and only applied strict pressure dressing for two days post-operatively to prevent related complications. No fluid collection related complications, wound healing impairments or need for re-operation for such local complications were present in any of our cases. Currently, there is no study that focuses on a direct comparison of a perineal drain usage and pressure dressing and international guidelines do not have a recommendation on this issue [5, 6].In a survey analysis administered to members of the Society of Genitourinary Reconstructive Surgeons, more than a quarter of reconstructive urologists reported that they routinely use perineal drain after urethroplasty. Authors of that survey analysis also indicated that evidence or even opinion-based consensus on drain use is lacking [4].

While there are no reports or evidence of whether to use a perineal drainage catheter or not in previous literature, our cases with absolute absence of any related complications suggest that pressure dressing is a safe and reliable method and perineal drain placement is unnecessary, even in the presence of complicating factors for wound healing such as smoking, obesity and history of pelvic radiation. Few of our patients had transient mild ecchymosis without collection around the incision site but no adjacent interventions were required. Also, we assume that subcutaneous ecchymosis would likely be present even perineal drain was inserted since it would drain inner layers and not efficiently expected to drain the subcutaneous area.

In addition to having possibly no usefulness in urethroplasty via perineal approach, drain placement may also be related with increased morbidity. Drains are known to cause discomfort, pain, infection and bleeding and even hematoma in drain sites. Although not directly relevant, in a study evaluating pain after retropubic radical prostatectomy, pain was found to be attributable drain placement in 24% of cases [7]. However, this number is likely to be much lower in perineal area since drain size would be lower and the muscle mass in insertion site is not comparable.

The main limitation of our study is its retrospective design and lack of comparison arms. Although our patient series with absence of any wound related complications makes a case, we agree that well-constructed comparison studies between drain usage and applying only pressure dressing are necessary to obtain higher level evidence and establish a peri-operative care management for urethroplasties. However, the absolute absence of complications that would be prevented with a drainage catheter suggests that drain is not necessary since we had no complications even without a drain. Applying pressure seems to be a safe method and perineal drain after urethroplasty a drain should not be routinely inserted, especially in uncomplicated cases.

## Conclusion

Our results indicate that applying pressure dressing is an effective method to prevent fluid collections and related morbidities, thus routine perineal drain may not be mandatory after urethroplasty surgeries by perineal approach.

## Data Availability

The data that support the findings of this study are not openly available due to reasons of sensitivity and are available from the corresponding author upon reasonable request. Data are located in Dokuz Eylul University Hospital storage.
